# Functional hepatobiliary organoids recapitulate liver development and reveal essential drivers of hepatobiliary cell fate determination

**DOI:** 10.1093/lifemedi/lnac055

**Published:** 2022-12-07

**Authors:** Juan He, Haoyue Cui, Xiaohan Shi, Qiqi Jin, Ximeng Han, Tiantian Han, Jiayin Peng, Shiwei Guo, Lei Zhang, Yun Zhao, Bin Zhou, Luonan Chen, Lei Chen, Yi Arial Zeng, Hongyang Wang, Gang Jin, Dong Gao

**Affiliations:** State Key Laboratory of Cell Biology, Shanghai Key Laboratory of Molecular Andrology, Shanghai Institute of Biochemistry and Cell Biology, Center for Excellence in Molecular Cell Science, Chinese Academy of Sciences, Shanghai 200031, China; University of Chinese Academy of Sciences, Beijing 100049, China; School of Life Science and Technology, ShanghaiTech University, Shanghai 201210, China; Department of Hepatobiliary Pancreatic Surgery, Changhai Hospital, Second Military Medical University, Shanghai 200433, China; School of Life Science and Technology, ShanghaiTech University, Shanghai 201210, China; State Key Laboratory of Cell Biology, Shanghai Key Laboratory of Molecular Andrology, Shanghai Institute of Biochemistry and Cell Biology, Center for Excellence in Molecular Cell Science, Chinese Academy of Sciences, Shanghai 200031, China; State Key Laboratory of Cell Biology, Shanghai Key Laboratory of Molecular Andrology, Shanghai Institute of Biochemistry and Cell Biology, Center for Excellence in Molecular Cell Science, Chinese Academy of Sciences, Shanghai 200031, China; State Key Laboratory of Cell Biology, Shanghai Key Laboratory of Molecular Andrology, Shanghai Institute of Biochemistry and Cell Biology, Center for Excellence in Molecular Cell Science, Chinese Academy of Sciences, Shanghai 200031, China; Department of Hepatobiliary Pancreatic Surgery, Changhai Hospital, Second Military Medical University, Shanghai 200433, China; State Key Laboratory of Cell Biology, Shanghai Key Laboratory of Molecular Andrology, Shanghai Institute of Biochemistry and Cell Biology, Center for Excellence in Molecular Cell Science, Chinese Academy of Sciences, Shanghai 200031, China; State Key Laboratory of Cell Biology, Shanghai Key Laboratory of Molecular Andrology, Shanghai Institute of Biochemistry and Cell Biology, Center for Excellence in Molecular Cell Science, Chinese Academy of Sciences, Shanghai 200031, China; State Key Laboratory of Cell Biology, Shanghai Key Laboratory of Molecular Andrology, Shanghai Institute of Biochemistry and Cell Biology, Center for Excellence in Molecular Cell Science, Chinese Academy of Sciences, Shanghai 200031, China; State Key Laboratory of Cell Biology, Shanghai Key Laboratory of Molecular Andrology, Shanghai Institute of Biochemistry and Cell Biology, Center for Excellence in Molecular Cell Science, Chinese Academy of Sciences, Shanghai 200031, China; The International Cooperation Laboratory on Signal Transduction, Eastern Hepatobiliary Surgery Hospital, Second Military Medical University, Shanghai 200438, China; National Center for Liver Cancer, Shanghai 200441, China; State Key Laboratory of Cell Biology, Shanghai Key Laboratory of Molecular Andrology, Shanghai Institute of Biochemistry and Cell Biology, Center for Excellence in Molecular Cell Science, Chinese Academy of Sciences, Shanghai 200031, China; The International Cooperation Laboratory on Signal Transduction, Eastern Hepatobiliary Surgery Hospital, Second Military Medical University, Shanghai 200438, China; National Center for Liver Cancer, Shanghai 200441, China; Department of Hepatobiliary Pancreatic Surgery, Changhai Hospital, Second Military Medical University, Shanghai 200433, China; State Key Laboratory of Cell Biology, Shanghai Key Laboratory of Molecular Andrology, Shanghai Institute of Biochemistry and Cell Biology, Center for Excellence in Molecular Cell Science, Chinese Academy of Sciences, Shanghai 200031, China

**Keywords:** liver development, cell lineage plasticity, hepatobiliary organoids

## Abstract

During liver development, hepatocytes, and cholangiocytes are concurrently differentiated from common liver progenitor cells and are assembled into hepatobiliary architecture to perform proper hepatic function. However, the generation of functional hepatobiliary architecture from hepatocytes *in vitro* is still challenging, and the exact molecular drivers of hepatobiliary cell lineage determination is largely unknown. In this study, functional hepatobiliary organoids (HBOs) are generated from hepatocytes. These HBOs contain a bile duct network surrounded by mature hepatocytes and stably maintain hepatic characteristics and function *in vitro* and upon transplantation *in vivo*. Morphological transition and expression profile of hepatocyte-derived organoids recapitulate the process of liver development. Gene regulation landscape of hepatocyte-derived organoids reveal that *Tead4* and *Ddit3* promote the cell fate commitment of liver progenitors to functional cholangiocytes and hepatocytes, respectively. Liver cell fate determination is reversed by inhibiting *Tead4* or increasing Ddit3 expression both *in vitro* and upon transplantation *in vivo*. Collectively, hepatocyte-derived HBOs reveal the essential transcription drivers of liver hepatobiliary cell lineage determination and represent powerful models for liver development and regeneration.

## Introduction

During liver development, hepatocytes, and cholangiocytes are derived from common liver progenitors. Hepatocytes could be de-differentiated into liver progenitor cells [1] and generate both hepatocytes and bile duct cells upon liver injury [2–9]. However, it remains elusive about the underlying mechanisms of liver hepatobiliary cell lineage determination during liver development and disease. These studies highlight the need to identify the essential drivers for cell fate determination during liver hepatobiliary formation.

In the liver, the bile canaliculus of hepatocytes are connected with cholangiocyte-derived bile ducts to secret bile [10]. Although, hepatocytes undertake the main function of liver, disorders of the biliary system including both intrahepatic and common bile duct account for 70% of pediatric and up to a third of adult liver transplantation. The disorders of the biliary system could lead the failure of liver transplantation [11]. This phenomenon further highlights that unique assembly of hepatobiliary architecture is essential for proper liver function [12]. However, recent progresses focused on the generation of functional hepatocytes [13–24] and cholangiocytes [25, 26] separately. Generation of functional hepatobiliary structures from adult liver cells is still a challenge *in vitro*. Therefore, the generation of functional hepatobiliary structures is urgently needed for liver regeneration and modeling liver development and disease.

Here, this study established a 3-dimensional (3D) hepatocyte culture system for generating self-assembled functional hepatobiliary organoids (HBOs) *in vitro*. These HBOs developed regional identities similar to adult liver hepatobiliary system with continuous bile duct networks surrounded by functional hepatocytes both *in vitro* and upon transplantation *in vivo*. Through integration of multiomics analysis of hepatocyte-derived organoids, we delineated the regulation network of the hepatobiliary cell lineage plasticity and identified Ddit3 and Tead4 as potential master transcription drivers for the cell fate determination of hepatocytes and cholangiocytes during liver hepatobiliary formation.

## Results

### The generation of HBOs from hepatocytes

To isolate hepatocytes, we used *Hnf4a-Dre*^ER^; *R26-RSR-tdTomato* mouse model to specifically label hepatocytes in mouse liver [27], and sorted tdTomato^ + ^hepatocytes for organoid culture (Fig. S1A and S1B). The purity of the sorted tdTomato^ + ^cells was over 99.5% (Fig. S1C and S1D). TdTomato^ + ^hepatocytes were successfully cultured and formed solid organoids, termed as solid hepatocyte organoids (SHOs) (Fig. S1E and S1F). SHOs transformed to hollow organoids after 4 passages of culture. These hollow organoids were termed as hollow hepatocyte organoids (HHOs). HHOs were highly proliferative and could be stably passaged for over 25 times (Fig. S1G–S1I).

**Figure 1.**
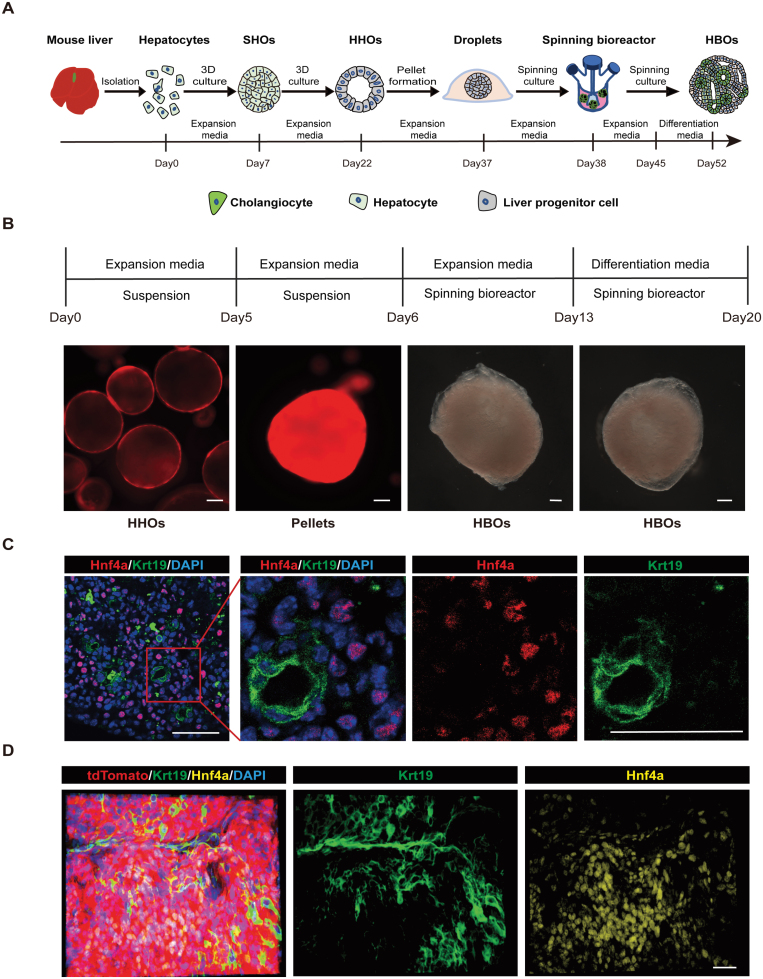
**Generation of functional hepatobiliary organoids.** (A) Schematic of the hepatobiliary organoid formation. Hepatocytes firstly formed SHOs and then transformed into HHOs. HHOs (over P10) were used to form the pellets by 96-well suspension plate. Then the pellets were embedded with Matrigel and transported into bioreactor to expand for 7 days and differentiated for 7 days to generate HBOs. (B) The representative image of organoids at different stage during hepatobiliary organoid formation. (C) Immunofluorescence staining of HBOs with hepatocyte marker Hnf4a and cholangiocyte marker Krt19. (D) Whole-mount immunofluorescence staining of the HBOs with Hnf4a and Krt19. Scale bars: 50 µm.

A variety of *in vitro* model systems have demonstrated that forced cell aggregation induced the self-organization of induced pluripotent stem cells (iPSCs) to form organ like tissues [28–32] and the forced cell aggregates could enhance the hepatic differentiation of liver buds [31]. To generate functional hepatobiliary structure, we collected 100,000 cells from HHOs and forced these cells to form a cell pellet using centrifuge. Then, the cell pellets were embedded with Matrigel and transferred to a spinning bioreactor to enhance nutrient absorption, providing the environment for intrinsic self-organization and differentiation (Fig. 1A and 1B). Strikingly, the differentiated pellets in bioreactor could self-assembled into complex structures containing Krt19 positive duct structures surrounded with Hnf4a and Alb positive hepatocytes (Figs. 1B, 1C and S2A). We termed these differentiated pellets as HBOs. Moreover, whole-mount staining of these HBOs showed that the Krt19 positive cells were connected as networks, resembling the intrahepatic bile duct-like structures (Figs. 1D and S2B; Video 1). Collectively, our culture system allowed hepatocytes rapid proliferation and generated liver organoids recapitulating the hepatobiliary structure of liver tissue.

### HBOs exhibit the essential signature and function of liver hepatobiliary structures *in vitro*

**Figure 2.**
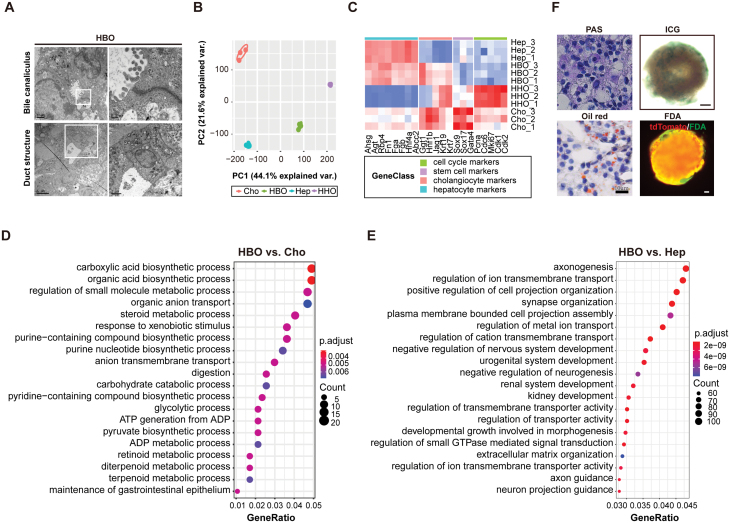
**HBOs exhibit the characteristics and function of both hepatocytes and cholangiocytes.** (A) Transmission EM (TEM) of HBOs shows typical hepatocyte and bile duct structures. (B) PCA plot of HBOs, HHOs, Hep, and Cho; Hep: primary hepatocytes, Cho: primary cholangiocytes. (C) Heatmap of representative markers in HBO, HHO, Hep, and Cho. (D) GO analysis of the differentiated expression genes between HBOs and cholangiocytes. (E) GO analysis of the differentiated expression genes between HBOs and hepatocytes. (F) Liver function analyses were performed in HBOs with PAS staining, oil red staining, ICG or FDA uptake, Scale bars for result of ICG and FDA uptake: 50 µm.

We next investigated whether HBOs exhibited the characteristics and function of hepatobiliary cells. The bile canaliculi structure indicated the establishment of epithelial polarization and was essential for hepatocyte function. Ultrastructural analysis with electron microscopy revealed the existence of both hepatocytes and bile duct structures in HBOs (Fig. 2A). The bile canaliculi structures were formed in the intercellular space of adjacent hepatocytes in HBOs (Fig. 2A, the upper panel). Moreover, we found that HBOs had binucleated cells which is a typical characteristic of mature hepatocytes (Fig. S3A). These results were consistent with the immunostaining results that Krt19^ + ^duct cells were surrounded by Hnf4a^+^/Alb^ + ^hepatocytes (Figs. 1C and S2A).

Consistently, gene expression profiles showed that HBOs had characteristics of both hepatocytes and cholangiocytes (Fig. 2B and 2C). To test whether these HBOs were bipotential liver progenitor cells, we examined the expression of proliferative and stem cell markers in HBOs. HBOs enriched with mature hepatobiliary signatures showed low expression level of liver progenitor markers (Fig. 2C). HBOs were enriched with basic hepatocyte function including lipid metabolism, glucose metabolism, and Cyp450s (Figs. 2D and S3B–G). HBOs were also enriched with the function of cholangiocytes including the pathways of transport regulation (Fig. 2E). GSEA analysis indicated that HBOs showed the enrichment of cholangiocyte-regeneration regulation pathway ERBB signaling pathway [33] (Fig. S3H). Moreover, PAS, oil red staining, Indocyanin green (ICG), and fluorescein diacetate assay (FDA) uptake results verified that the mature HBOs had the typical liver function (Fig. 2F). Collectively, HBOs had both typical characteristics and essential function of liver hepatobiliary structures.

### HBOs reconstitute the hepatobiliary structures *in vivo*

**Figure 3.**
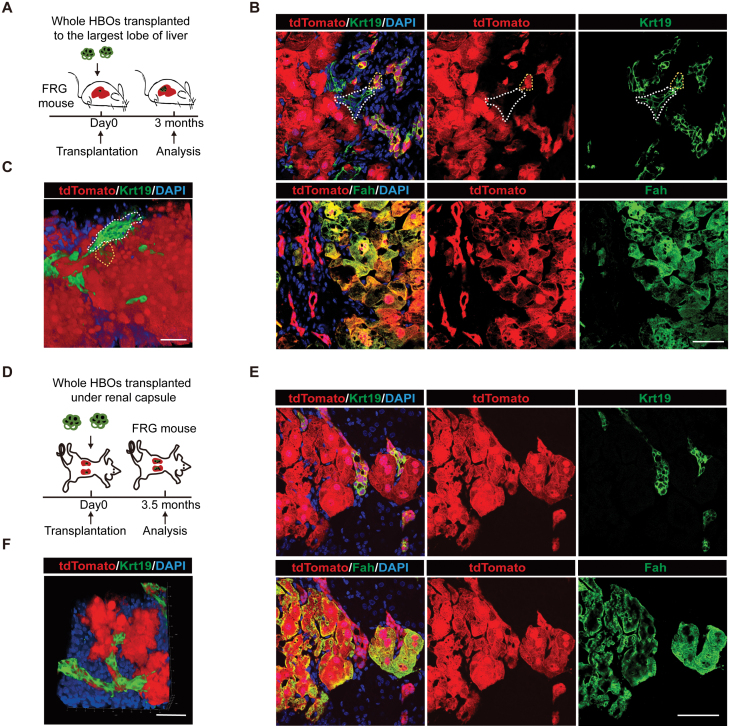
**HBOs generate hepatobiliary structures upon orthotopic and ectopic transplantation.** (A) Schematic of strategy to research the function of HBOs orthotopically. The HBOs were transplanted into the liver of FRG mice as a whole and analyzed 3 months after transplantation. (B) Immunofluorescence staining of HBO-transplanted FRG mouse liver with hepatocyte marker Fah and cholangiocyte marker Krt19. White dash line indicated the recipient bile duct. Yellow dash line indicated HBO-derived bile duct. (C) Whole-mount immunofluorescence staining of the FRG mouse liver transplanted with HBOs with Krt19. White dash line indicated the recipient bile duct. Yellow dash line indicated HBO-derived bile duct. (D) Schematic of strategy to research the function of HBOs ectopically. The HBOs were transplanted under renal capsule of FRG mice as a whole and analyzed after transplanted for 3.5 months. (E) Immunofluorescence staining of HBOs-transplanted FRG mouse kidney with Fah and Krt19. (F) Whole-mount immunofluorescence staining of HBOs-transplanted FRG mouse kidney with Krt19. Scale bars: 50 µm.

Next, we investigated whether HBOs could form the functional hepatobiliary structures *in vivo.* We transplanted whole HBOs into the largest lobe of FRG mouse liver (Fig. 3A). We traced the transplanted HBOs by tdTomato *in vivo* and found that HBOs expanded in FRG mouse livers 3 months after HBOs transplantation (Fig. S4A). HBOs generated both Alb^+^/Hnf4a^+^/Fah^ + ^hepatocytes and Krt19^ + ^cholangiocytes (Figs. 3B and S4B). Remarkably, we found that the HBO-derived cholangiocytes formed the bile duct networks which were connected with the recipient bile ducts (Fig. 3B and 3C; Video 2). We repeated these transplantations over three times and the results were consistent.

To explore whether HBOs reconstitute the functional hepatobiliary structure ectopically, we transplanted HBOs under renal capsule of FRG mice (Fig. 3D). Excitedly, we observed that HBOs expanded under renal capsule for 3.5 months (Fig. S4C). More importantly, the transplanted HBOs generated both hepatocytes and cholangiocytes under renal capsule (Figs. 3E and S4D). And the HBO-derived cholangiocytes also formed the bile duct networks surrounded by hepatocytes (Fig. 3F; Video 3). We repeated the renal capsule transplantation of HBOs over three times and the results were similar. In collection, these results demonstrated that HBOs were able to generate the hepatocytes and bile duct system orthotopically and ectopically *in vivo*.

### SHOs, HHOs, and HBOs recapitulate the liver development process

**Figure 4.**
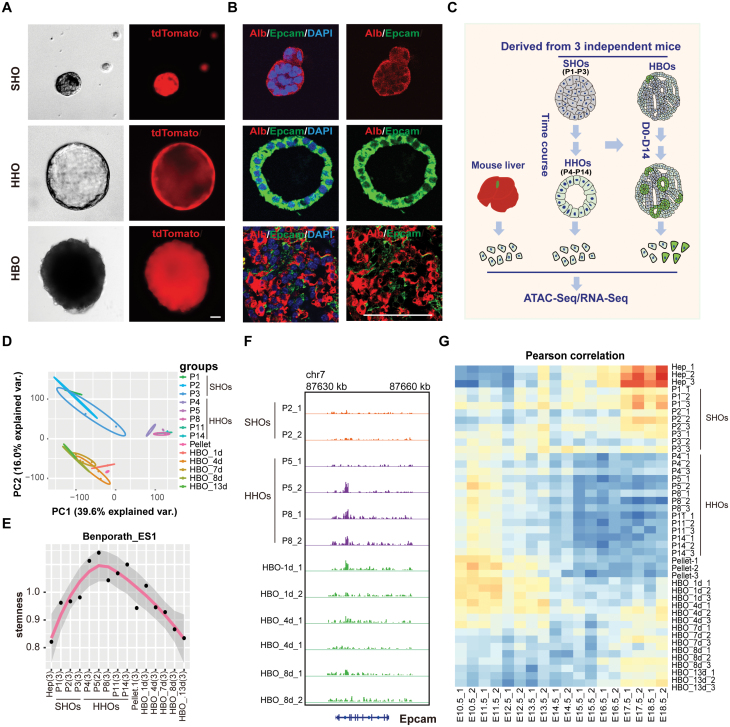
**SHOs, HHOs, and HBOs recapitulate the plasticity of hepatocytes and liver development.** (A) Bright-field and tdTomato fluorescence images of hepatocyte organoids at different stage demonstrated the change of organoid shape. SHOs (P1–P3) were solid structure, HHOs (P4–P14) were hollow structure and HBOs were solid structures. (B) Immunofluorescence staining of SHOs, HHOs, and HBOs with hepatocyte marker Alb and peogenitor/cholangiocyte marker Epcam. (C) Experimental strategy to analyze the transcription network during the process from SHOs to HHOs and to HBOs with RNA-Seq and ATAC-Seq. (D) PCA plot of SHOs, HHOs, and HBOs with RNA-Seq data. The hepatocytes were divided into three group: SHOs, HHOs, and HBOs. (E) Stemness change of SHOs, HHOs, and HBOs. (F) IGV snapshot showing the open peak in Epcam locus between SHOs, HHOs, and HBOs. (G) Similarity of SHOs, HHOs, and HBOs with fetal liver samples from E10.5 to E18.5. Scale bars: 50 µm.

During the hepatocyte culture process, we found that hepatocyte-derived organoids underwent a shape switch from SHOs to HHOs (Fig. S5A). Correspondingly, upon transforming from SHOs to HHOs, the organoid cells lost the expression of hepatocyte marker Alb and gained the expression of progenitor/cholangiocyte marker Epcam. Strikingly, the process from HHOs to HBOs underwent a shape switch from hollow structure to solid hepatobiliary structure (Fig. 4A and 4B). Epcam expression and hollow structure of organoids are considered as the characteristics of liver stem cells [34]. These organoid shape changes recalled the process of liver bud initiation and liver development that also have the morphological transition from hollow structure to solid tissue [35].

To characterize the dynamic transcriptional change of SHOs, HHOs, and HBOs, we performed RNA-Seq and ATAC-Seq (Fig. 4C). In line with the change of organoid shape and gene expression (Fig. 4A and 4B), the assay of RNA-Seq data and ATAC-Seq data showed that SHOs, HHOs, and HBOs were separated from each other and HBOs were similar to SHOs (Figs. 4D, S5B and S5C) Besides, the stemness increased during the process from SHOs to HHOs and decreased from HHOs to HBOs. HHOs showed highest stem cell capacity among SHOs, HHOs, and HBOs (Figs. 4E and S5D). Furthermore, increased chromatin accessibility of stemness marker *Epcam* was identified in HHOs compared with SHOs and HBOs (Fig. 4F).

To further analyze whether SHOs, HHOs, and HBOs could recapitulate the liver tissue profiles during development, we compared SHOs, HHOs, and HBOs with mouse liver cells at different development stages [36]. Notably, SHOs showed high similarity with the differentiated liver cells around embryonic day 18.5 (E18.5). Consistent with the highest stem cell activity, HHOs was highly correlated with the liver progenitor cells around E10.5 (Fig. 4G). The process from HHOs to HBOs showed the similar gene expression pattern of liver development from E10.5 to E18.5 (Figs. 4G and S5E). Therefore, HHOs, SHOs, and HBOs provided a powerful model to recapitulate the state of liver stem cell and differentiated liver cells during development.

### Identification of the regulatory network of liver cell fate determination

Transcription factors (TFs) have been recognized as the master drivers of tissue development and cell fate determination [37]. Then, we investigated the transcription regulators of the liver cell lineage determination with regulon analysis using SHOs, HHOs, and HBOs models. We modified the single-cell regulatory network inference and clustering (SCENIC) pipeline [38] and integrated the filtered regulators from RNA-Seq analysis with the enriched motif by the corresponding ATAC-Seq data to measure regulon activities (Fig. S6A and S6B). We finally found a total of 116 regulons as potential master TFs in regulating liver cell fate determination and liver development.

**Figure 5.**
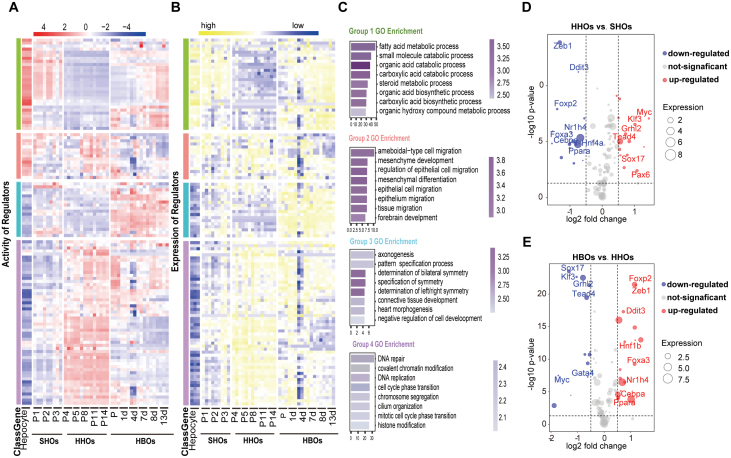
**Identification of the regulation network of liver cell fate decision.** (A and B) The activity (A) and expression (B) level of regulons during the process. (C) GO analysis of gene sets regulated by regulons of different groups. (D) Volcano plot of the differential active regulon between SHOs and HHOs. Blue represented TFs that were down-regulated in HHOs, red was up-regulated TFs in HHOs, and gray indicated TFs that do not change significantly during the process. (E) Volcano plot of the differential active regulons between HHOs and HBOs. Blue represented TFs that were down-regulated in HBOs, red is up-regulated TFs in HBOs, and gray indicates TFs that do not change significantly during the process.

These regulons could be divided into four groups based on their activity or expression (Fig. 5A and 5B). Group 1 regulons (G1 regulons) were enriched in SHOs and HBOs, including classic hepatocyte TFs, such as *Hnf4a*, *Ppara*, *Cebpa,* and *Nr1i2* (Fig. S6C). As expected, G1 regulon-target genes were enriched in the typical hepatic function pathways (Figs. 5C and S6C). Meanwhile, HBOs also had its specific regulon group G3 including *Hnf1b* that was associated with bile duct cell development (Figs. 5A and S6E). *Hnf1b* was known as master transcription driver of bile duct cell fate determination. Consistently, cholangiocytes emerged during HBO formation. G2 regulon did not show a specific distribution during the samples and they mainly regulated tissue development and migration processes according to the GO analysis of their regulated genes (Figs. 5C and S6D). G4 regulons including *Gata4, Sox9,* and *Sox17* related to early liver development [39] were enriched in HHOs (Fig. S6F). Genes regulated by G4 regulon were enriched with pathways mainly regulating the proliferation-related process including cell cycle, DNA replication, and histone modification (Fig. 5C).

To further characterize the master drivers in regulating liver cell fate determination at progenitor stage (HHOs) or mature stage (SHOs and HBOs), we performed differential assay to find the significantly enriched regulons at different stage (Fig. 5D and 5E). Compared with HHOs, most of the enriched regulons in SHOs were also enriched in HBOs (Fig. S6G, upper panel). The up-regulated regulons during the de-differentiation process (SHOs to HHOs) were down-regulated during the differentiation stage (HHOs to HBOs) (Fig. S6G, down panel). These results indicated that the overlapped regulons potentially regulated the liver cell fate determination during liver development.

### Tead4 and Ddit3 are potential master TFs for hepatobiliary cell fate determination

**Figure 6.**
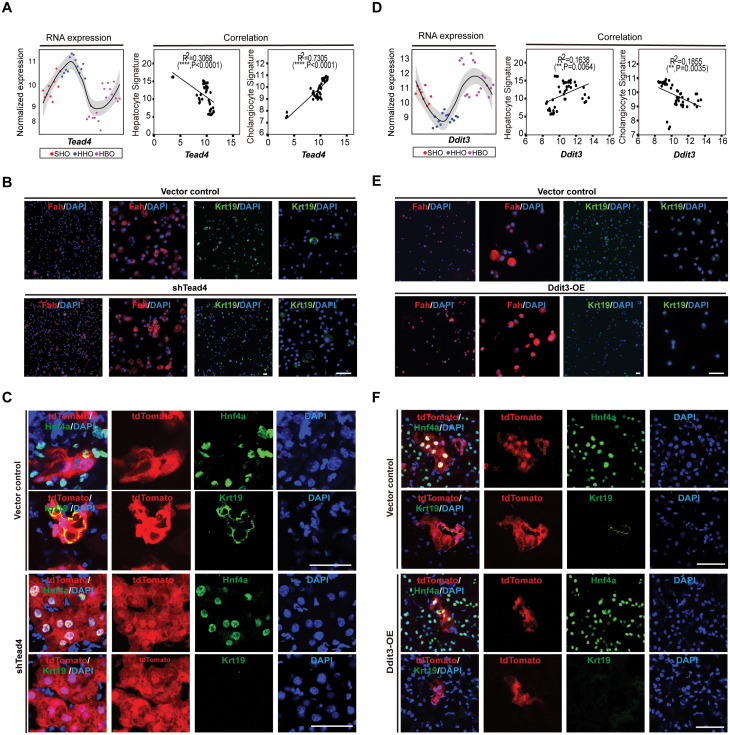
**Tead4 and Ddit3 positively regulate the cell-fate decision of cholangiocytes and hepatocytes, respectively.** (A) The expression level of *Tead4* and its correlation with the expression of hepatocyte signature and cholangiocyte signature. (B) Immunofluorescence staining of the Tead4-knockdown HHOs after differentiation for 7 days with Fah and Krt19. (C) Immunofluorescence staining of the Tead4-knockdown cell-derived renal capsule grafts with Hnf4a and Krt19. (D) The expression level of *Ddit3* and its correlation with the expression of hepatocyte signature and cholangiocyte signature. (E) Immunofluorescence staining of the Ddit3-overexpression HHOs after differentiation for 7 days with Fah and Krt19. (F) Immunofluorescence staining of the Ddit3-overexpression cell-derived renal capsule grafts with Hnf4a and Krt19. Scale bars: 50 µm.

To further define the regulators of cell fate determination during hepatobiliary formation, we overlapped the significantly changed regulons from SHOs to HHOs and from HHOs to HBOs. We then ranked them by integrating both FDR value and activity foldchange (Table S1) and further investigated the top four regulators enriched in progenitor stage (HHOs). *Tead4*, *Myc*, *Klf3,* and *Grhl2* were enriched in HHOs and positively correlated with cholangiocyte signature and negatively correlated with hepatocyte signature (Figs. 6A and S7A). These regulators may play essential roles in cholangiocyte fate determination during hepatobiliary formation.

Knockdown of *Tead4* significantly increased the expression of hepatocyte marker gene Alb and inhibited the expression of cholangiocyte marker gene Krt19 (Figs. 6B, S7B and S7C). Knockdown of *Tead4* also enhanced the differentiation of hepatocytes in HBO formation (Fig. S7D). Moreover, knockdown of *Tead4* blocked the formation of cholangiocytes after transplantation under renal capsule of FRG mouse (Figs. 6C and S7E). In contrast with the phenotype of Tead4-knockdown, overexpression of Tead4 enhanced the expression of cholangiocyte marker gene Krt19 and inhibited the expression of hepatocyte marker gene Fah (Fig. S7F and S7G). Consistent with previous work, these results confirmed that Tead4 play an important role in cholangiocyte regeneration [1, 40].

We also investigated top four regulators enriched in mature stage (SHOs and HBOs). By contrast, the identified regulators *Ddit3*, *Zeb1*, *Foxa3,* and *Foxp2* were positively correlated with hepatocyte signature and negatively correlated with cholangiocyte signature (Figs. 6D and S8A). Next, we overexpressed *Zeb1*, *Ddit3*, *Foxa3,* and *Foxp2* in HHOs (Fig. S8B). Among them, overexpression of Ddit3 significantly enhanced the expression of hepatocyte marker genes (Fah, Alb) and inhibited the expression of cholangiocyte marker genes (Krt19) in both RNA and protein levels (Figs. 6E and S8C). Moreover, Ddit3 overexpression inhibited the formation of cholangiocytes and generated hepatocytes in HBO formation or after transplantation under renal capsule of FRG mouse (Figs. 6F and S8D). And losing function of Ddit3 inhibited the expression of hepatocyte marker gene Fah and increased the expression of cholangiocyte marker gene Krt19 (Fig. S8E and S8F). In collection, we identified Tead4 and Ddit3 as master drivers of cholangiocytes and hepatocytes determination during hepatobiliary formation, respectively.

## Discussion

Hepatobiliary system is the fundamental architecture for the hepatic function. Many efforts have been made to generate the liver organoids containing single cell type including hepatocytes or cholangiocytes. These organoids can be derived from adult stem cells or iPSC and are helpful to purely study the function and behavior of hepatocytes or cholangiocytes. To model liver tissue or investigate the function and development of hepatocytes and cholangiocytes concurrently, formation of HBOs containing both cell types are needed. The generation of functional hepatobiliary structure is essential for liver regeneration both *in vitro* and upon transplantation *in vivo*. iPSC-derived endoderm cells combined with endothelial cells and mesenchymal progenitors generated functional liver bud *in vivo* [31, 41]. However, the *in vitro* culture system to generate hepatobiliary structure is still needed. Although co-differentiation of hepatocyte-like cells and cholangiocyte-like cells from iPSCs successfully generated the HBOs, other germ layers were also generated as byproducts during the process [42]. Besides, fetal liver progenitor cells combined with the liver extracellular matrix produced hepatocytes with bile duct-like structures [43]. And combing hepatocytes and cholangiocytes was able to generate the liver organoids with hepatobiliary connection [44]. However, the functional HBOs derived from adult hepatocytes were still limited.

Here, we have established a novel hepatocyte-derived HBO system specialized with 3D and bioreactor suspension culture. First, with 3D culture, mature hepatocytes were de-differentiated into highly proliferative liver progenitor cells. Second, although bioprinting or biomanufacturing is able to provide the scaffold for cell growth and cell patterning to form the functional complex structures, these systems also introduce the extra materials and are not flexible to perform high-throughput parallel experiments. We used spinning bioreactor to provide flow suspension environment, enhance the nutrient absorption, and promote the self-assembly of progenitors into substantial functional HBOs. These derived HBOs showed both the characteristics and function of liver hepatobiliary cells and generated the hepatobiliary structures upon transplantation *in vivo.* Third, this system recapitulated the process of liver development and provided an *in vitro* model to identify the master TFs for liver cell fate determination.

**Figure 7.**
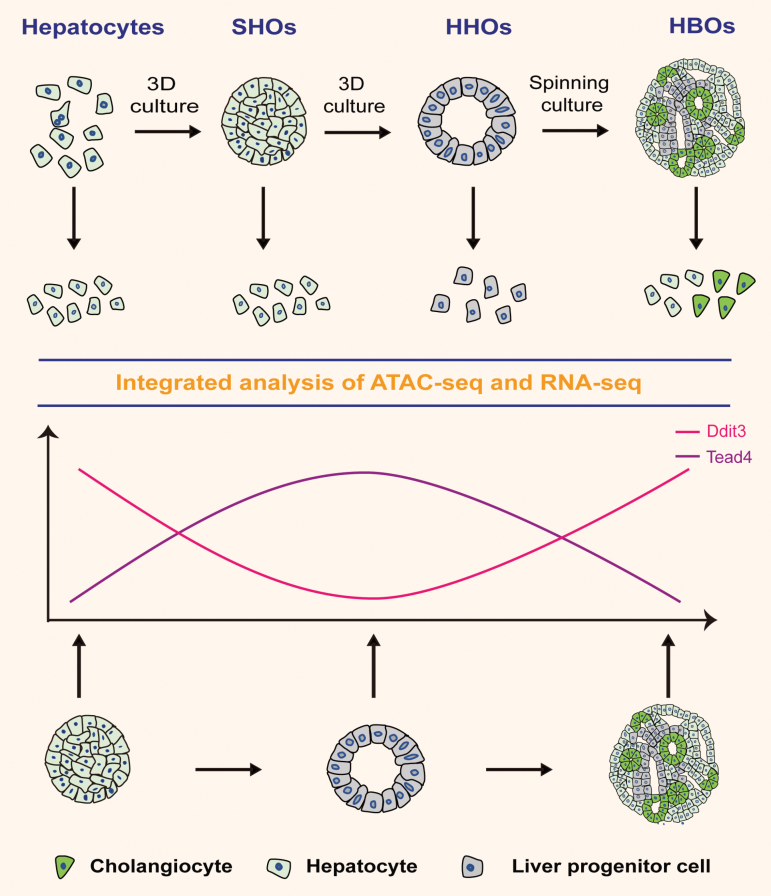
**Graphic summary of liver organoid culture systems for liver development and liver regeneration research.** The liver organoid culture system of this study allowed the proliferation of mouse hepatocytes and delineated the process from SHOs to HHOs and HBOs. The multiomics analysis of SHOs, HHOs, and HBOs identified Ddit3 and Tead4 as the potential TFs for regulating the cell fate determination of hepatocytes and cholangiocytes during hepatobiliary formation.

Hepatocytes showed great cell lineage plasticity to form liver progenitor cells and bile duct cells *in vivo* [1–7]. However, the underlying mechanisms were largely unknown. In addition, its ability to form the hepatobiliary structures simultaneously *in vitro* was poorly characterized. Previously, hepatocytes, and cholangiocytes were separately generated from hepatocyte-derived progenitors [21]. Here, we provided a culture system to simultaneously generate both hepatocytes and cholangiocytes. The culture system recapitulated the plasticity of hepatocytes from mature hepatocytes to progenitors and from progenitors to mature hepatobiliary cells. Furthermore, we performed the integrated ATAC-Seq and RNA-Seq to identify master transcription regulators of hepatobiliary cell fate determination during liver development. The identified regulators included the previous reported TFs essential for liver development such as *Hnf4a*, *Sox9,* and *Sox17* [45–49]. Besides, we identified potential master transcription drivers *Tead4* and *Ddit3* for the commitment of hepatocytes and cholangiocytes during hepatobiliary formation and reinforced the mutually exclusive relationship between hepatocytes and cholangiocytes (Fig. 7). These results inferred that inhibition of cholangiocyte differentiation were able to enhance the hepatocyte differentiation and vice versa.

In conclusion, this study advances the avenue to using mature hepatocytes to generate the HBOs *in vitro*, uncovers the master TFs in regulating liver hepatobiliary cell fate determination and provides a potential platform for studies of liver development, liver regeneration medicine, and liver diseases.

## Research limitations

This study also has several limitations. Although, this culture system successfully generated mouse hepatocyte-derived HBOs, the ability of human liver cells to form HBOs needs to be further studied. Besides, perturbation of Ddit3 and Tead4 have been investigated to influence the formation of hepatocytes and cholangiocytes during hepatobiliary cell fate determination. To dissect the underlying mechanism, more experiments including ATAC-seq and ChIP-seq after perturbation of Ddit3 or Tead4 are needed. Moreover, apart from Ddit3 and Tead4, the contribution of other identified regulons to the process of de-differentiation/re-differentiation remains be investigated. Finally, combination of cell barcode and single cell RNA-seq may be helpful to decipher the exact cell subpopulation involved in the process of de-differentiation and re-differentiation.

## Methods

### Mice

All mouse studies are approved by Animal Care and Use Committee of the Center for Excellence in Molecular Cell Science, Chinese Academy of Sciences. Mice were bred and maintained according to Shanghai Laboratory Animal Center Institutional Animal Regulations. *Hnf4a-Dre*^ER^; *R26-RSR-tdTomato* mice was generated and genotyped as previously described [27]. Fah^−/−^Rag2^−/−^Il2rg^−/−^ (FRG) triply mutant mice and NOD/SCID mice were maintained in specific pathogen-free husbandry. FRG mice were fed with 4 mg/mL 2-(2-nitro-4-trifluoro-methyl-benzoyl)-1,3 cyclohexanedione as drinking water. Generation and genotyping of FRG mice have been previously described [50].

### Mouse primary hepatocytes isolation and culture

For mouse hepatocytes, adult *Hnf4a-IRES-Dre*^*ER*^*; R26-RSR-tdTomato* mice after tamoxifen administration were subjected to standard three-step collagenase perfusion for isolation of primary hepatocytes. Briefly, the liver was pre-perfused through the inferior vena cava with calcium-free buffer (0.5 mM EGTA, 1 × EBSS without Ca2^+^ and Mg2^+^) and then perfused with calcium-buffer (10 mM HEPES, 1 × EBSS with Ca^2+^ and Mg^2+^), at last changed to collagenase type IV (0.25 mg/mL, Sigma) (10 mM HEPES, 1 × EBSS with Ca^2+^ and Mg^2+^) to digest tissue. Parenchymal cells were purified by Percoll (Sigma) buffer (90% Percoll, 1 × EBSS without Ca^2+^ and Mg^2+^) at low-speed centrifugation (1000 rpm, 5 min). Viability of isolated hepatocytes were around 95% as determined by Trypan blue staining. Mouse hepatocytes were mixed with Matrigel and 1000 hepatocytes in 50μL Matrigel drops were seeded in 24-well suspension culture plate. Culture medium was added until matrigel solidified. Culture media was based on Advance DMEM/F12 media supplemented with 10 mM HEPES (Gibco, USA), 2 mM GlutaMAX-1 (Gibco, USA), 500Xprimocin (InvivoGen, USA), 1 × B27 (Gibco, USA), 1.56 mM N-Acetylcysteine (Sigma, Germany), 0.5 μM A83-01 (Tocris, USA), 10 μM Y27632 (Selleck, USA), 50 ng/mL EGF (Peprotech, USA), 10 mM Nicotinamide (Sigma), 10 ng/mL FGF10 (Peprotech), 1 ng/mL FGF2 (Peprotech), homemade R-Spondin1 (2%), and Noggin (10%) conditioned media. Passage was performed every 4–7 days at a 1:4–1:8 split ratio for at least 8 months.

To prepare frozen stocks, organoids were dissociated and mixed with freezing medium (Gibco, USA), and then frozen with standard procedures. When required, the organoids were thawed using standard thawing procedures and cultured as described above.

### Mouse HBOs formation

To generate liver tissue *in vitro* with hepatobiliary structure, hepatocyte organoids beyond passage 10 (HHOs) were used. In total, 100,000 cells were plated in each well of an ultra-low-binding 96-well plate (Corning) and were spun at 500 g for 5 min to form pellet. After cultured for 1 day, the pellets were embedded with Matrigel and transferred to a spinning bioreactor (Wheaton, USA) for another 7 days. Subsequently, the HBOs were exposed to hepatic maturation media: Advance DMEM/F12 (Invitrogen) supplemented with 10 mM HEPES (Gibco, USA), 2 mM GlutaMAX-1 (Gibco, USA), 500X primocin (InvivoGen, USA), 1 × B27, 1.56 mM N-Acetylcysteine, 10 μM Y27632, 10 μM DAPT (Selleck), 25 ng/mL BMP7 (Peprotech), 25 ng/mL HGF (Peprotech), 3 μM Dexamethasone (R&D Systems), and 20 ng/mL oncostatin M (Wako) for 1 week in spinning bioreactor and developed a defined liver hepatobiliary structure.

### Quantitative real-time PCR (qRT-PCR)

RNA was isolated and purified using RNeasy Mini Kit (Qiagen, Germany) according to the manufacturer’s instructions. RNA concentration and purity were determined using NanoDrop-2000 spectrophotometer (Thermo Fisher Scientific). First-strand reverse transcription was performed with 0.5–1.0 µg of RNA using the PrimeScript RT Master Mix (Takara, Japan) as the manufacturer’s standard protocol. Gene expression analysis was carried out using QuantiNova SYBR Green RT-PCR Kit (Qiagen) on BioRad CFX96 Real-Time System. Gene transcription was evaluated using the ∆ Ct method normalized to Actin. Primer sequences and sources are listed in Table S2.

### Immunohistochemistry and immunofluorescence

Tissue paraffin embedding was performed as standard protocol. Organoid paraffin embedding was conducted by using a previously described method [51]. Paraffin-embedded tissues or organoids were cut into 4 μm sections and stored at room temperature, frozen tissues or organoids were cut into 10 μm sections and stored at −30°C refrigerator before staining. Paraffin-embedded samples were deparaffinized and underwent antigen retrieval in sodium citrate buffer (pH 6.0) in a steamer for 15 min. Sections were blocked and permeabilized in 1XPBS with 5% goat serum and 0.1% Triton X-100. Sections were then incubated with primary antibodies in 5% goat serum at 4°C overnight. For immunofluorescence, sections were incubated with appropriate secondary antibodies for 1 h at room temperature in the dark. Nuclei were stained with DAPI (Sigma). For whole-mount staining, we referred the reported method previously [52]. For immunohistochemistry, samples were incubated with appropriate secondary antibody (Zsbio, China) and performed as manufacturer’s instructions. The primary antibodies used as follows: goat anti Alb (Bethyl Laboratories, A90-134A, 1:500 dilution), rabbit anti Hnf4a (Cell Signaling Technology, 3113, 1:500 dilution), rat anti Krt19 (DSHB, TROMA-III, 1:500 dilution), mouse anti β-Catenin (Cell Signaling Technology, 37447, 1:200 dilution), rabbit anti FAH (Cell Lab Tech, CLT-602-910, 1:500 dilution). The secondary antibodies used for immunofluorescence are as follows: Cy3-conjugated donkey anti-goat IgG 1:1000 (Jackson Lab, USA), FITC-conjugated donkey anti rabbit IgG (Jackson Lab), FITC-conjugated donkey anti mouse IgG (Jackson Lab), Alexa fluor 488 goat anti mouse IgG (Life Technology), Alexa fluor 488 goat anti rabbit IgG (Life Technology), Alexa fluor 594 goat anti rabbit IgG (Life Technology), Alexa fluor 594 goat anti mouse IgG (Life Technology), Alexa fluor 647 goat anti rat IgG (Life Technology).

### Liver functional assays

The PAS staining system was purchased from Sigma-Aldrich and conducted according to the manufacturer’s instructions. Oil red staining was manipulated as user manual (Sigma-Aldrich). ICG uptake assay was performed by adding 1 mg/mL ICG (Sigma) in the medium for 30 min. For FDA uptake, HBOs were incubated 5 min at 37°C with 5 mg/mL FDA (Thermo Fisher) followed by PBS washing and taken for fluorescence imaging.

### Generation of stable cells using lentiviral infection

To knockdown mouse *Tead4*, *Klf3*, *Grhl2, Myc*, and *Ddit3*, four shRNAs were selected, respectively and cloned to the constitutively expressed vector SGEP as previously reported [53]. For lentiviral production, the lentiviral expression vector was co-transfected with the third-generation lentivirus packing vectors into 293T cells using VigoFect reagent. Then, 48–72 h after transfection, mouse hepatocyte cells were stably infected with viral particles and selected with 4 μg/mL puromycin. The knockdown-efficiency was analyzed by qPCR and at least two shRNAs were selected for the following function analysis, respectively. For overexpression, the coding domain sequence of Ddit3, Zeb1, Foxa3, and Foxp2 was cloned into PCDH vector and using lentiviral infection to generate the stable cell lines. Tead4 ovexpression plasmids were kindly from the laboratory of Lei Zhang.

### Western blot

Western blotting was done as previously described (Zhang et al., 2015). The following primary antibodies were used: Flag (LS-C331459, LifeSpan BioSciences) and β-actin (54979, Cell Signaling Technology).

### ATAC-Seq library construction

Mouse hepatocyte organoids at different passage were collected and dissociated with TrypLE to prepare single cell suspension. Around 50,000 cells were loaded for the following library construction with the optimized protocol to reduce contaminating mitochondrial DNA [54]. Briefly, cells were washed once with PBS and then lysed in 50 μL ice-cold lysis buffer (10 mM pH 7.4 Tris-HCl; 10 mM NaCl; 3 mM MgCl_2_; 0.1% NP-40; 0.1% Tween-20; 0.01% digitonin) for 3 min on ice and were immediately washed with 1 mL wash buffer (10 mM pH 7.4 Tris-HCl; 10 mM NaCl; 3 mM MgCl2; 0.1% Tween-20) and then spun at 500 g for 10 min at 4°C. To generate sequencing libraries, TruePrep DNA Library Prep Kit V2 for Illumina (Vazyme, TD501) was utilized for following steps.

### RNA-Seq library construction and analysis

Total RNA was isolated from hepatocytes in different passage, HBOs and primary hepatocytes and cholangiocytes. To get primary hepatocytes, 3-steps perfusion was used to dissociate hepatocytes which have been illustrated detailly in the section of Mouse primary hepatocytes isolation and culture. For cholangiocyte isolation, the mouse liver tissue was cut into small pieces which were digested with type ║ collagenase in 37°C. After dissociated into single cell suspension, the cells were incubated with Epcam-APC-Cy7 (Biolegend,118218, 1:500) antibody on ice for 20 min, stained with DAPI and then isolated with flow cytometry sorting to enrich Epcam positive live cells. The hepatocytes and cholangiocytes were isolated into the TRIzol without culture to avoid changing remarkable expression changes. RNA sequencing libraries were prepared with the VAHTS mRNA-seq V3 Library Prep Kit for Illumina (Vazyme, NR611). Sequencing was performed by Berry Genomics (China). Low-quality sequences and adapters were filtered by Trimmomatic. Clean reads were mapped to the grcm38 genome using hisat2-2.1.0. Expression was quantified at the gene level using stringtie v1.3.3. Differentially expressed genes (DEGs) were analyzed by DESeq2 using counts. And *P* value < 0.05 was set as threshold to define DEGs. Enriched pathways analysis was performed by enrichGO and cluster Profiler with the annotation data set parameter setting at Reactome pathways. GSEA-2.2.4 tool was used to identify enriched transcriptome signatures using normalized expression value generated by DESeq2. Analysis was performed with c2-cp-reactome.v6.2.symbols as gene sets database. Number of permutations were set at 1000 and other basic and advanced fields were set to default. The gene sets for stemness analysis were basically from previous studies [55, 56]. ssGSEA of GSVA R package was used to calculate the stemness of the hepatocyte-derived organoids. To compare with the fetal liver data [36], the gene sets were acquired by the common genes expressed in both data sets and followed by Pearson correlation coefficient calculation.

### ATAC-Seq analysis

ATAC-seq data were processed against the mm10 reference genome and aligned by using BWA. Peaks were called by using MACS2, filtered to *q* value < 0.01. Peaks and differential sites were visualized by using IGV, counts were generated for each peak set as input by using bedtools-multicov and normalized. Differential binding cluster analysis was performed by using R-DiffBind. Motif enrichment was analyzed by using MEME CentriMo, all sequences lengths are 500-bp as input files, HOCOMOCO Mouse (v11 FULL) dataset as the reference.

### Regulon analysis

SCENIC was used to identify the regulons based on gene expression as the previous study reported [38] and after integrated with the enriched motif from ATAC-Seq, the activity of filtered regulon of each biological replicate was calculated at each period. For volcano plot, the average expression level of regulon expression was subtracted to represent size of the dots. The regulon activity of the two periods was divided and changed to the log formation to represent Abscissa. About vertical coordinates, *F* test was performed for regulon activity over two periods, *t* test for genes whose activity followed the distribution of *F*, or Welch *t* test for otherwise. Fdr correction was performed on the *P* value, and changed to log formation.

### Transplantation

All animal experiments in the current study, including experimental procedures, sample isolation, and animal care were approved and in accordance with the guidelines and regulations from Animal Care and Use Committee of the Center for Excellence in Molecular Cell Science, Chinese Academy of Sciences. For liver transplantation, around 15 HBOs without breaking or TrypLE digestion were transplanted into the largest lobe of FRG mouse liver. For ectopic transplantation, 10–15 HBOs for one kidney were transplanted under renal capsule of FRG mouse as previously reported [57]. All animal experiments were performed in accordance with institutional regulations.

### Statistics

All data are presented as mean ± s.e.m. For qRT-PCR assay etc. statistical evaluation, an unpaired Student’s *t*-test was used to calculate statistical probability in this study. Statistical calculation was performed using Graphpad software. For all statistics, data from at least three independent experiments were used.

## Data availability

The raw data that support the findings of this study are available from The National Omics Data Encyclopedia (NODE) OEP003725.

## Supplementary data

Supplementary material is available at *Life Medicine* online.

Video 1. Whole mount staining of HBOs with Krt19.

Video 2. Whole mount staining of HBO-derived liver graft with Krt19.

Video 3. Whole mount staining of HBO-derived renal capsule graft with Krt19.

Table S1. Regulon activity in hepatocyte-derived organoids (SHO, HHO, and HBO).

Table S2. Primers used for gene expression analysis in this study.

lnac055_suppl_Supplementary_Figures

lnac055_suppl_Supplementary_Table_S1

lnac055_suppl_Supplementary_Table_S2

lnac055_suppl_Supplementary_Video_S1

lnac055_suppl_Supplementary_Video_S2

lnac055_suppl_Supplementary_Video_S3
