# Supplementary material for: Functional hepatobiliary organoids recapitulate liver development and reveal essential drivers of hepatobiliary cell fate determination
Source: Life Med. 2022 Dec 7;1(3):345–58. doi: 10.1093/lifemedi/lnac055 (PMC11749142; doi:10.1093/lifemedi/lnac055)
Supplement: lnac055_suppl_Supplementary_Figures [file lnac055_suppl_Supplementary_Figures.docx]

**Functional hepatobiliary organoids recapitulate liver development and reveal essential drivers of hepatobiliary cell fate determination**

**Supplementary figures**

**
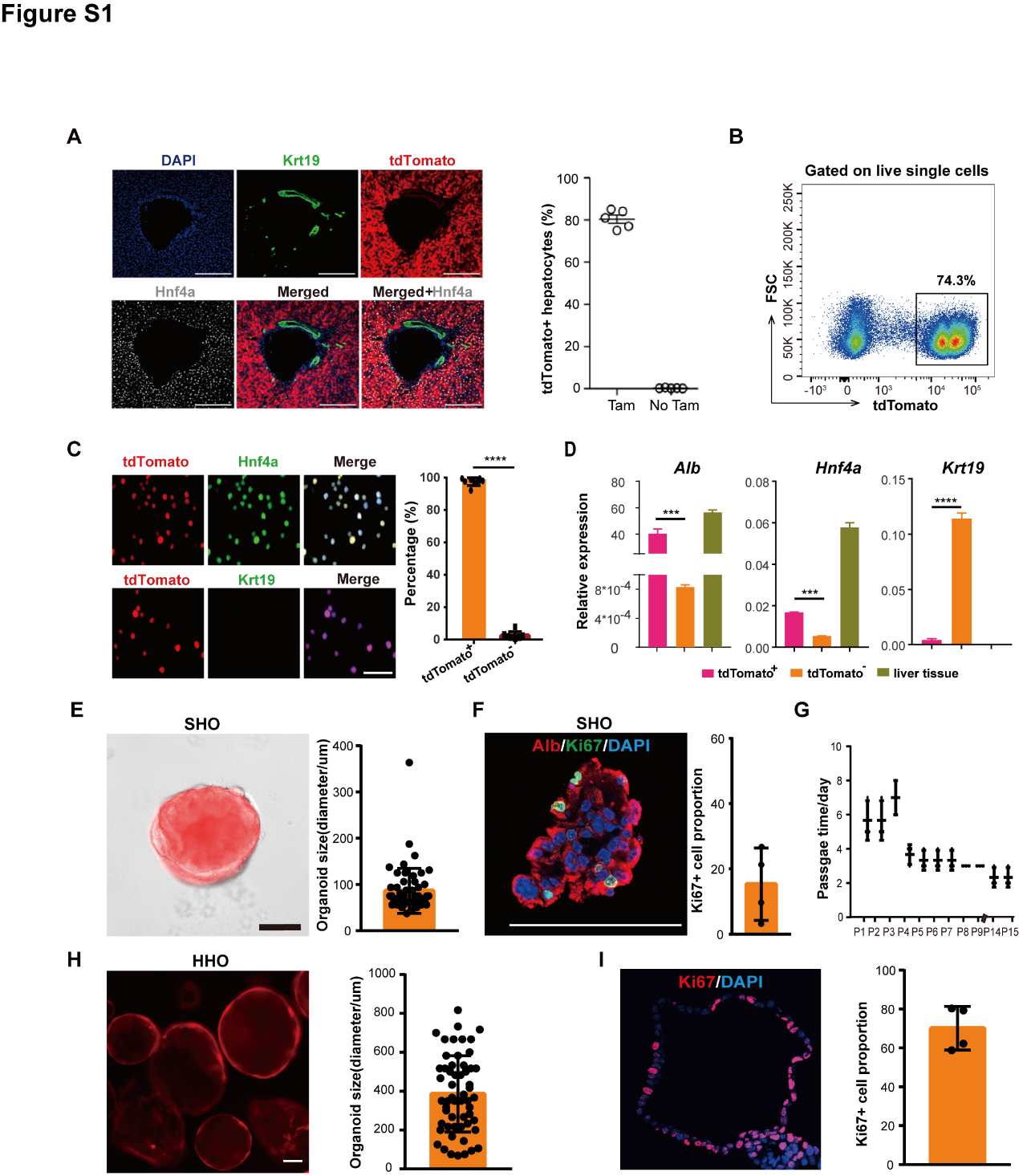
**

**Figure S1. Long-term culture of hepatocytes.** (**A**) Immunostaining for tdTomato, Krt19 and Hnf4a of liver sections showed that Hnf4a-Dre^ER^ specifically labeled 80% hepatocytes and could not label cholangiocytes. (**B**) Purification of hepatocytes by FACs sorting tdTomato positive cells of *Hnf4a-IRES-DreER; R26-RSR-tdTomato* mouse liver. (**C-D**) Identity of sorted hepatocytes are confirmed by immunofluorescence (**C**) and qRT-PCR (**D**). ****p* < 0.001 and *****p* < 0.0001 (two-tailed Student’s *t* test). The purity of tdTomato^+^ cells was over 99.5% (**C**). (**E**) Representative phase contrast and tdTomato fluorescent image of solid hepatocyte organoids (SHO) indicating that mature hepatocytes can form organoids *in vitro*. (**F**) Immunostaining for Alb and Ki67 of SHOs showed that Alb positive hepatocytes were proliferative under the culture system. (**G**) The time course of passage indicated that the hepatocytes could be cultured for a long term and HHOs proliferated faster than SHOs. (**H**) Representative phase contrast and tdTomato fluorescent image of HHOs (after P4) showed that the organoids were hollow. (**I**) Immunostaining for Ki67 of HHOs showed that HHOs were highly proliferative. Scale bars: 50 µm.


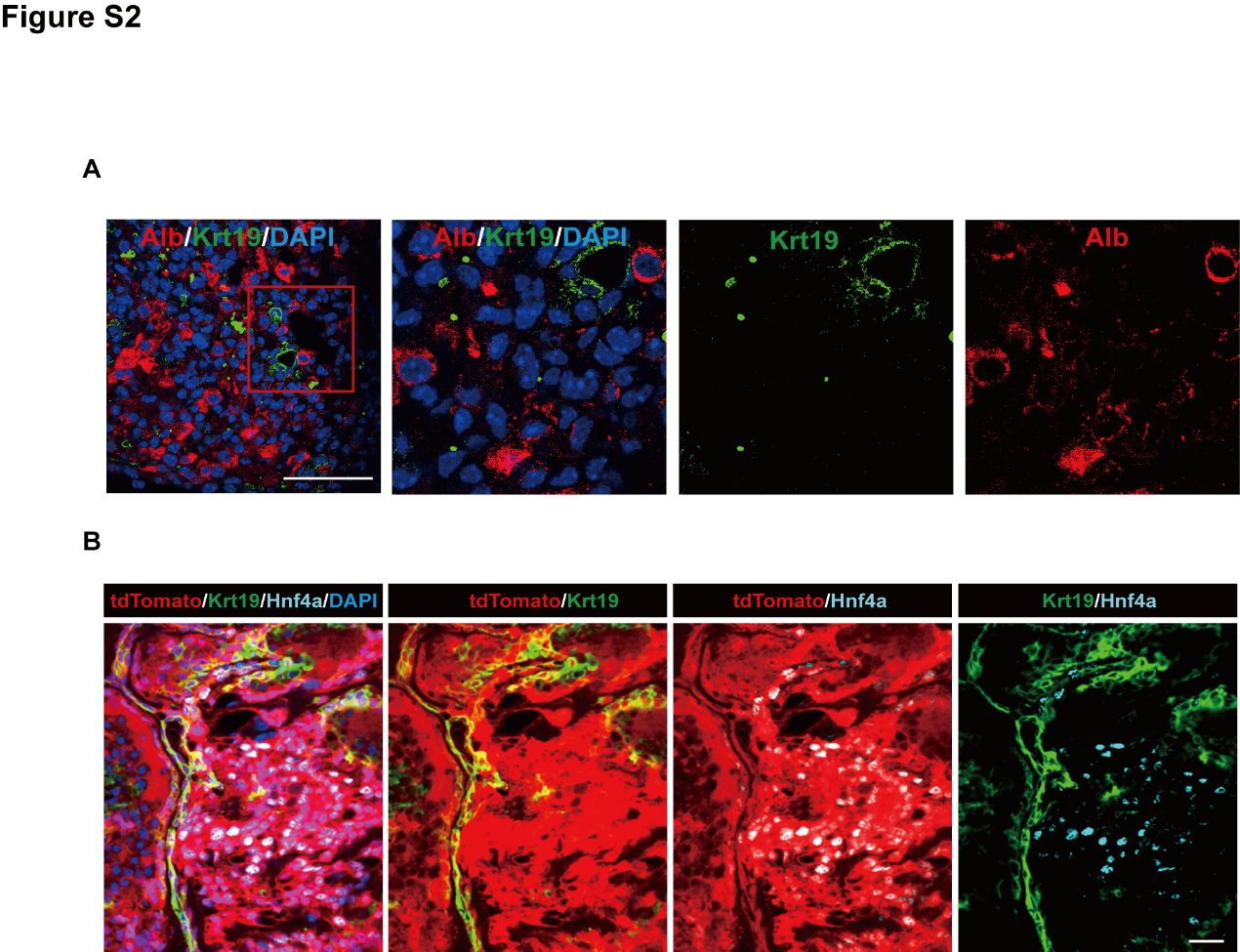


**Figure S2. Generation of functional hepatobiliary organoids.** (**A**) Immunostaining for Alb and Krt19 of hepatobiliary organoids showed that the organoids had Krt19 positive duct structures surrounded by Alb positive hepatocytes. (**B**) The section of whole mount staining of Hnf4a and Krt19 indicated that tdTomato positive cells contained both Krt19 positive cells and Hnf4a positive cells. Scale bars: 50 µm.


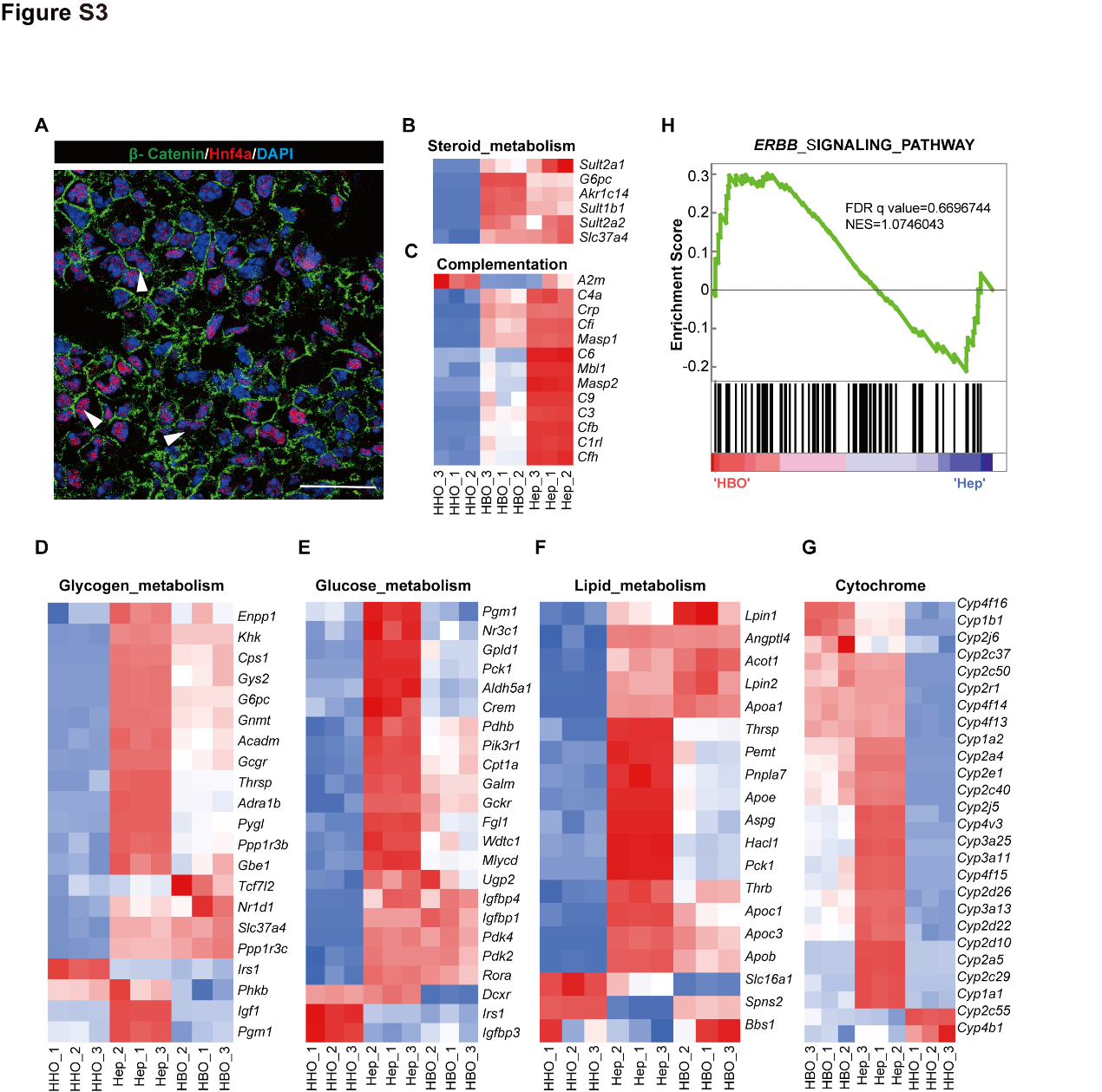


**Figure S3. Identification of the function and characteristics of hepatobiliary organoids.** (**A**) Immunostaining for β-catenin and Hnf4a of hepatobiliary organoids. The arrowhead showed Hnf4a positive binucleate hepatocytes. (**B-G**) Heatmaps comparing HBOs (*n* = 3) with primary hepatocytes (*n* = 3) and HHOs (*n* = 3) in steroid metabolism (**B**), complement activation (**C**), glycogen metabolism (**D**), glucose metabolism (**E**), lipid metabolism (**F**) and Cytochrome activity (**G**). Gene set was basically from figure S2 (*1*). (**H**) GSEA analysis of the differentiated expression genes between hepatobiliary organoids and hepatocytes. Scale bars: 50 µm.


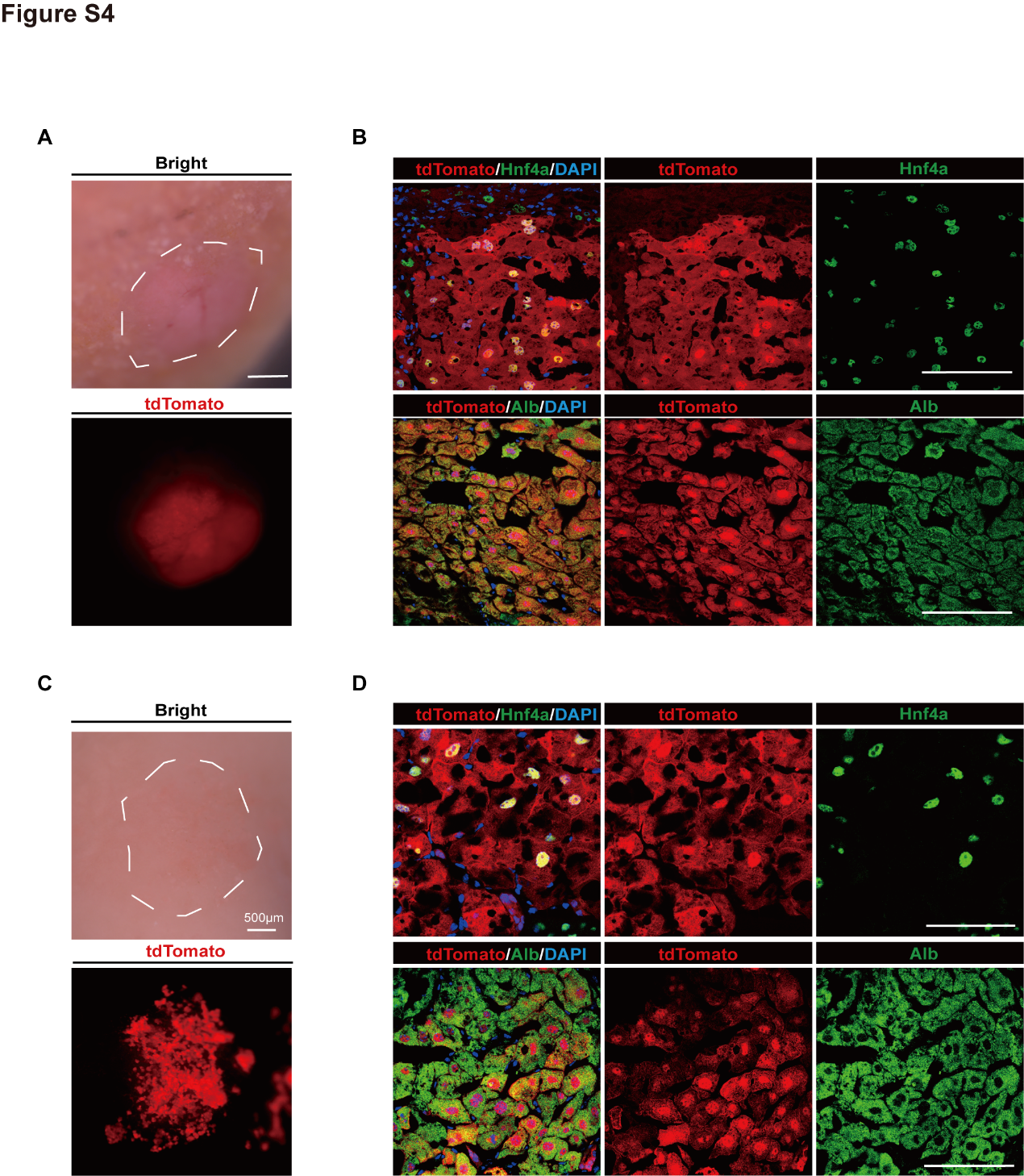


**Figure S4. Investigation of the function of hepatobiliary organoids by *in-vivo* transplantation.** (**A**) Representative bright field and tdTomato fluorescent image of HBO transplanted mouse liver. tdTomato positive field indicated the transplanted HBO-derived tissue. Dash line indicated the transplanted tissue. (**B**) Immunofluorescence staining of FRG mouse liver transplanted with HBOs with hepatocyte marker Alb and Hnf4a. (**C**) Representative bright field and tdTomato fluorescent image of HBO transplanted mouse kidney. tdTomato^+^ cells demonstrating the resident of HBO. Dash line indicated the transplanted tissue. (**D**) Immunofluorescence staining of FRG mouse kidney transplanted with HBOs with hepatocyte marker Alb and Hnf4a. Scale bars: 50 µm.

**
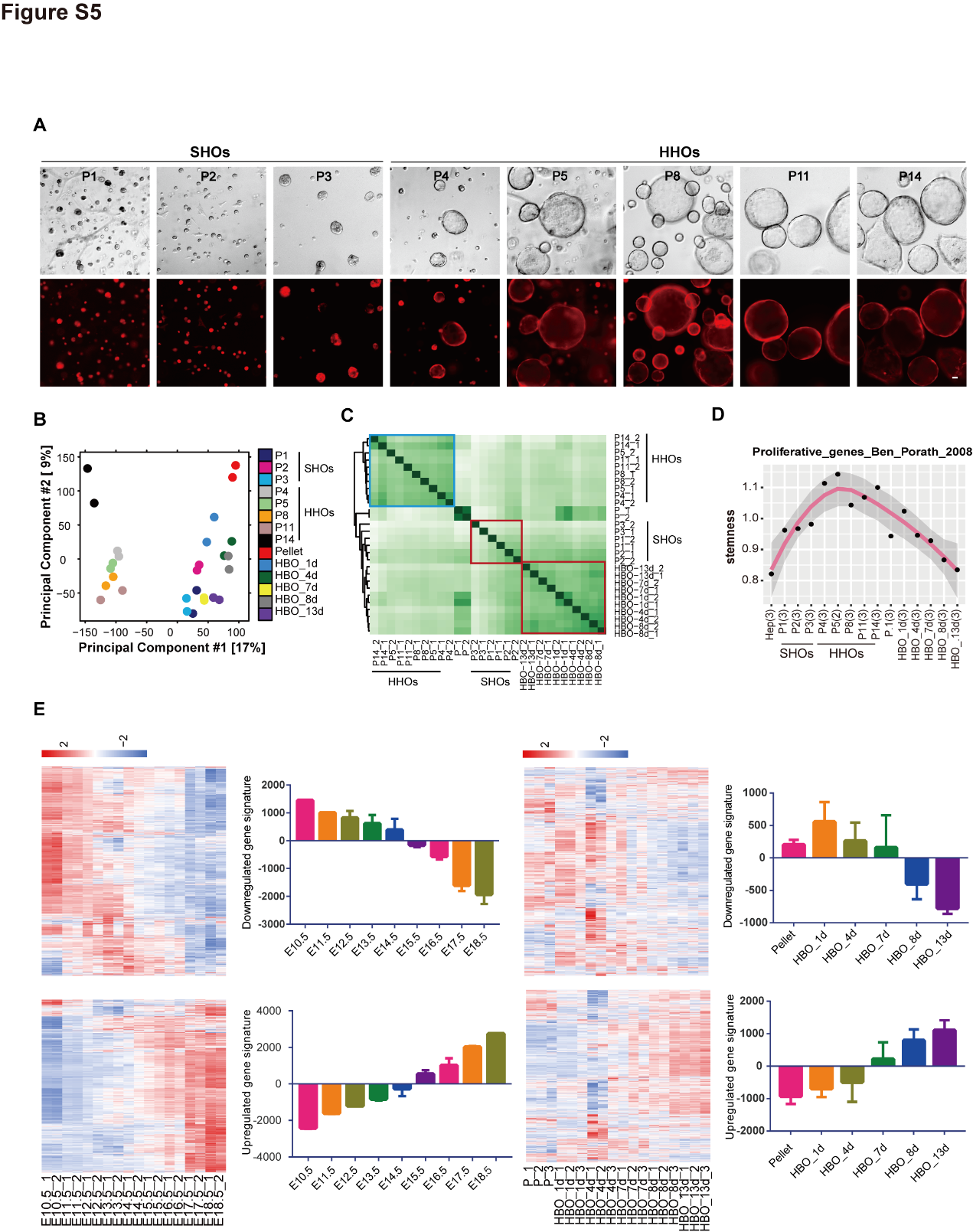
**

**Figure S5. The culture system of hepatocyte derived HBOs could recapitulate the plasticity of hepatocytes.** (**A**) Bright-field and tdTomato fluorescence images of hepatocyte organoids at different stage demonstrated the change of organoid shape. SHOs (P1-P3) were solid structure and HHOs (P4-P14) were hollow structure. (**B**) PCA plot of hepatocyte derived organoids at different stage with ATAC-Seq data. The HBOs are closer to the SHOs which is in lined with the result of RNA-Seq (Fig. 4D). (**C**) The correlation of hepatocytes at different stage by dba plot showed the same result with (**B**). (**D**) Stemness change of hepatocyte derived organoids at different stage. (**E**) Upregulated or downregulated gene sets in fetal liver samples showed the same expression pattern during the formation of hepatobiliary organoids. Data sets were basically from public literature (*2*) .Scale bars: 50 µm.


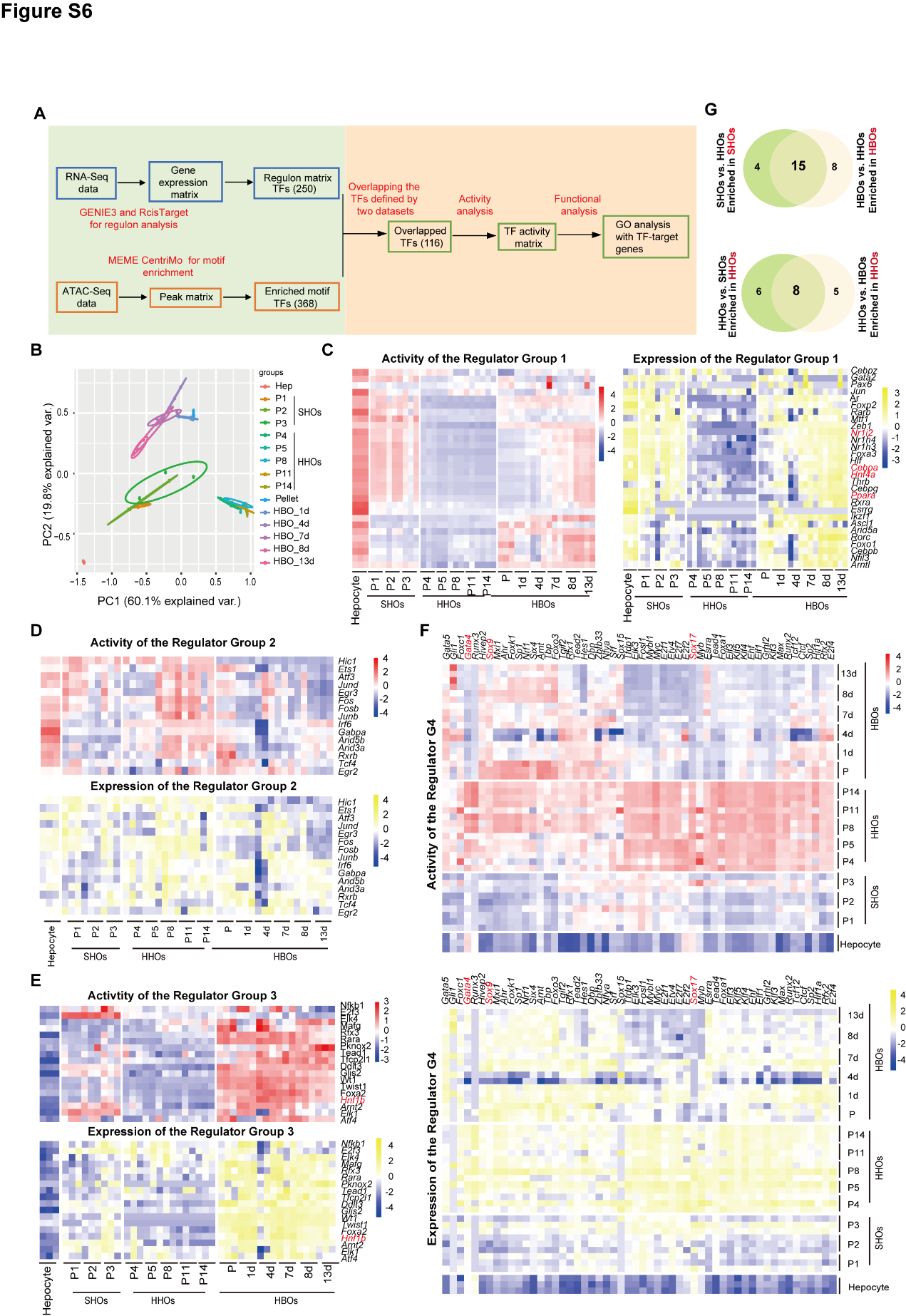


**Figure S6. Identification of the regulon network of liver cell fate decision between SHOs, HHOs and HBOs.** (**A**) Schematic of regulon analysis by integrating the RNA-Seq data and ATAC-Seq data of cells during culture. Datasets of transcription factors and co-expressed genes were obtained according to RNA-Seq, and motif networks that regulate gene expression were obtained by ATAC-Seq. The results of the RNA-Seq and ATAC-Seq were integrated to retain the overlapped transcription factors, and then the activity analysis of regulon and downstream analysis were performed. TFs: transcription factors. (**B**) The PCA plot of hepatocytes during culture with regulon activity which was in accordance with the result derived from all genes. (**C-F**) The activity and expression level of G1 regulons (**C**), G2 regulons (**D**), G3 regulons (**E**) and G4 regulons (**F**). The highlighted transcription factors with red color were reported transcription factors essential for regulating liver development. (**G**) Venn plot showing the overlapped regulators enriched in SHOs and HBOs after compared with HHOs in the upper panel. Venn plot showing the overlapped regulators enriched in HHOs after compared with SHOs or HBOs in the down panel.


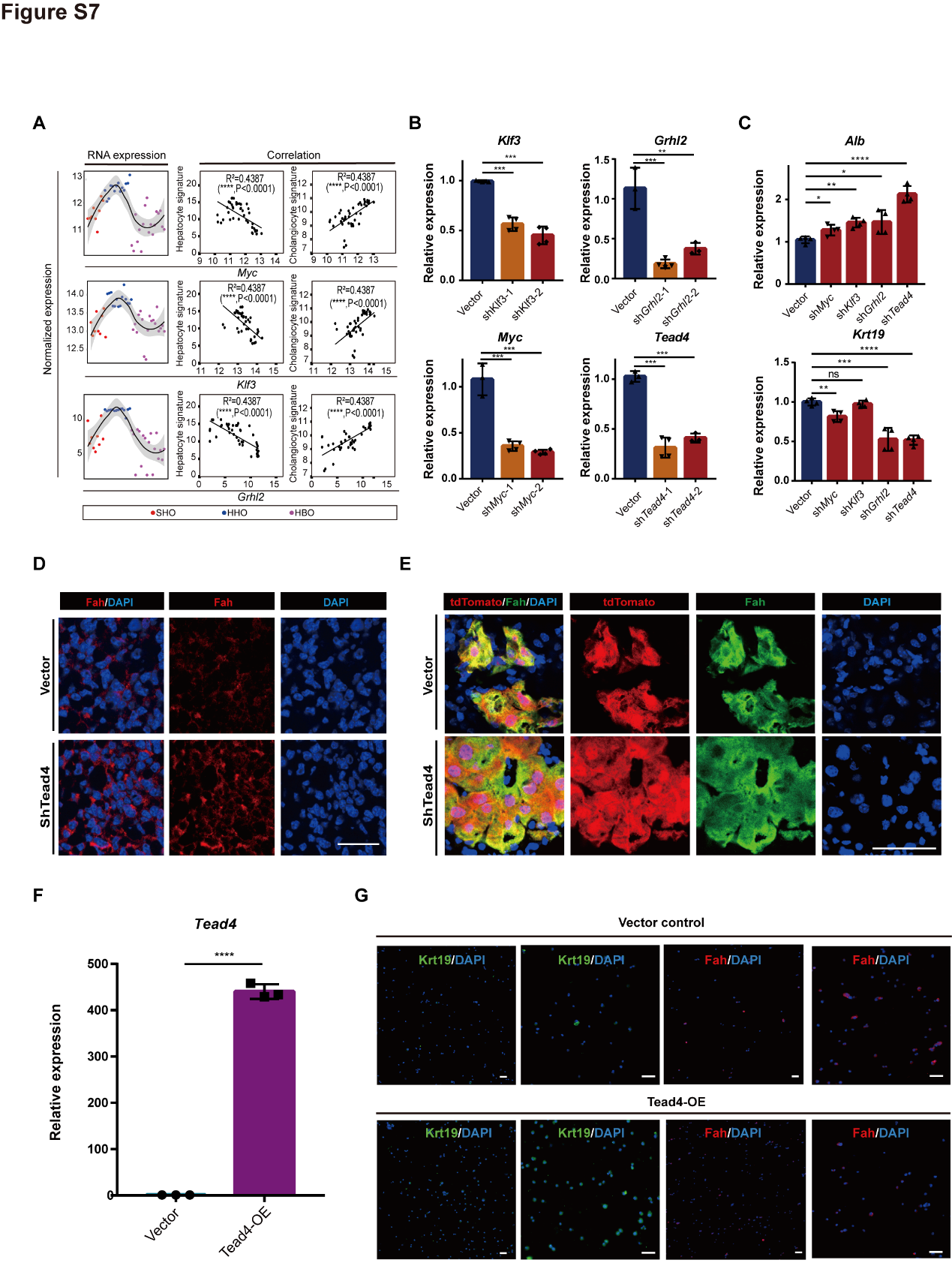


**Figure S7. Identifying the positive regulator of cholangiocyte fate decision.** (**A**) The expression level of *Klf3*, *Myc* and *Grhl2* and their correlation with the expression of hepatocyte signature and cholangiocyte signature. (**B**) RT-qPCR analysis of the knockdown efficiency of *Tead4*, *Klf3*, *Myc* and *Grhl2*. **p* < 0.05, ***p* < 0.01, ****p* < 0.001 and *****p* < 0.0001 (one-way ANOVA). Data are shown as mean ± SEM, *n* = 4. (**C**) RT-qPCR analysis of the *Tead4*, *Klf3*, *Myc* and *Grhl2*-knockdown progenitors after differentiation for 7 days with the hepatocyte marker *Alb* and cholangiocyte marker *Krt19*. Knockdown of *Tead4* could most strongly inhibit the expression of cholangiocyte marker *Krt19* and induce the expression of hepatocyte marker *Alb*. **p* < 0.05, ***p* < 0.01, ****p* < 0.001 and *****p* < 0.0001 (one-way ANOVA). Data are shown as mean ± SEM. (**D**) Immunofluorescence staining of the *Tead4*-knockdown HBOs with the hepatocyte marker Fah. (**E**) Immunofluorescence staining of the *Tead4*-knockdown renal capsule grafts with the hepatocyte marker Fah. Scale bars: 50 µm. (**F**) RT-qPCR of Tead4 to confirm Tead4 overexpression. *****p* < 0.0001 (two-tailed Student’s t test), *n* = 4 (**G**) Immunostaining of Fah and Krt19 in HHOs with or without Tead4 overexpression after differentiation. Scale bars: 50 µm.


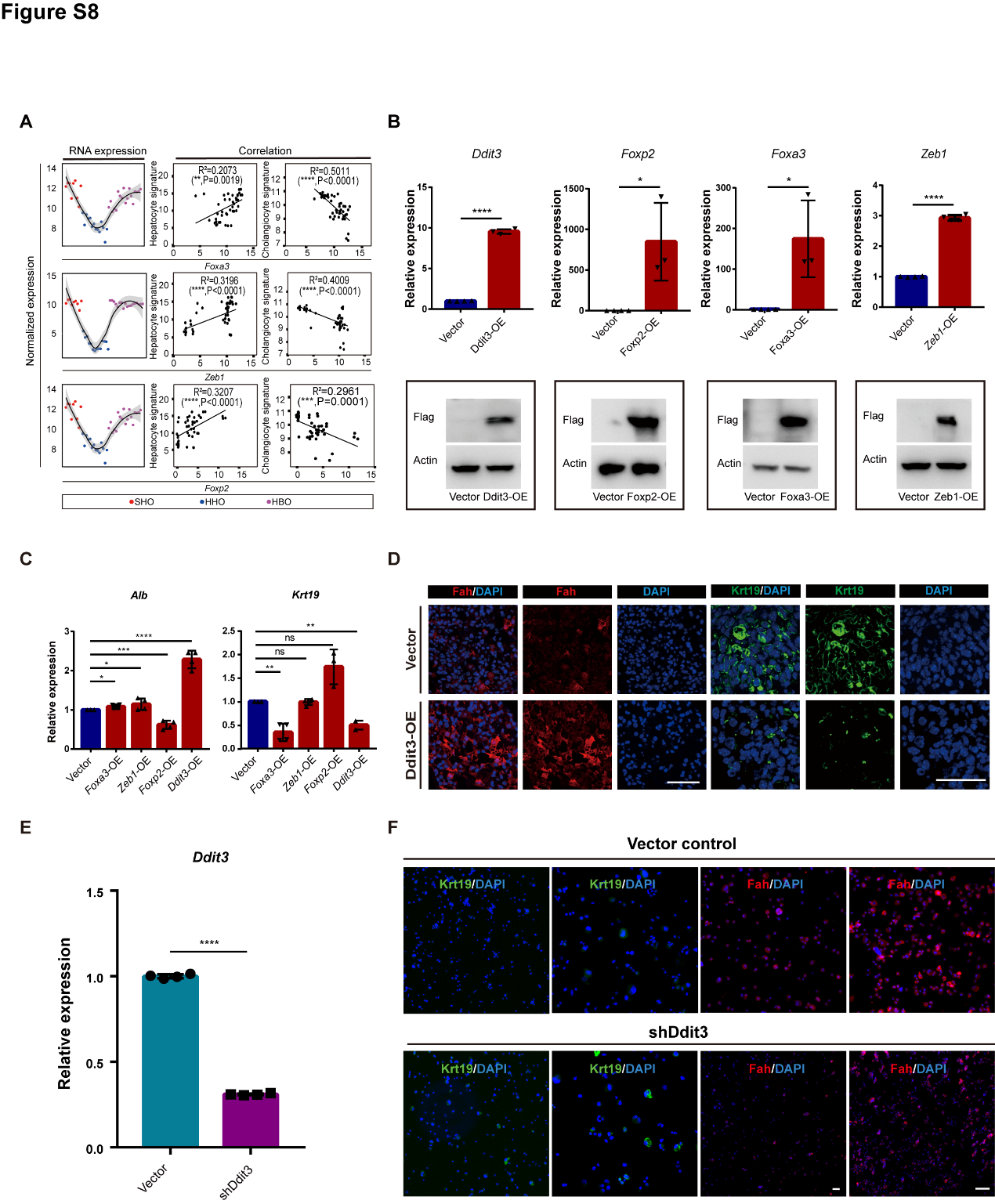


**Figure S8. Investigating the positive regulator of hepatocyte cell fate decision.** (**A**) The expression level of *Foxa3*, *Foxp2* and *Zeb1* and their correlation with the expression of hepatocyte signature and cholangiocyte signature. (**B**) RT-qPCR and western blot analysis of the overexpression efficiency of *Ddit3*, *Zeb1*, *Foxa3* and *Foxp2*. Data are shown as mean ± SEM, **p* < 0.05 and *****p* < 0.0001 (two-tailed Student’s *t* test), *n* = 4 (**C**) RT-qPCR analysis of the *Ddit3*, *Zeb1*, *Foxa3* and *Foxp2*-overexpression progenitors after differentiation for 7 days with the hepatocyte marker *Alb* and cholangiocyte marker *Krt19*. **p* < 0.05, ***p* < 0.01, ****p* < 0.001 and *****p* < 0.0001 (one-way ANOVA). Data are shown as mean ± SEM. (**D**) Immunofluorescence staining of the Ddit3-overexpression HBOs with the hepatocyte marker Fah and cholangiocyte marker Krt19. Scale bars: 50 µm. (**E**) RT-qPCR of Ddit3 to confirm Ddit3 knockdown. *****p* < 0.0001 (two-tailed Student’s t test), *n* = 4 (**F**) Immunostaining of Fah and Krt19 in HHOs with or without Ddit3-knockdown after differentiation. Scale bars: 50 µm.

**REFERENCES**

1. T. Katsuda *et al.*, Conversion of Terminally Committed Hepatocytes to Culturable Bipotent Progenitor Cells with Regenerative Capacity. *Cell Stem Cell* **20**, 41-55 (2017).

2. L. Yang *et al.*, A single-cell transcriptomic analysis reveals precise pathways and regulatory mechanisms underlying hepatoblast differentiation. *Hepatology* **66**, 1387-1401 (2017).
